# Development and Characterization of Nanobodies Targeting the Kupffer Cell

**DOI:** 10.3389/fimmu.2021.641819

**Published:** 2021-02-22

**Authors:** Fang Zheng, Jinhong Zhou, Zhenlin Ouyang, Jiaxin Zhang, Xinyi Wang, Serge Muyldermans, Jo Van Ginderachter, Nick Devoogdt, Yurong Wen, Steve Schoonooghe, Geert Raes

**Affiliations:** ^1^The Key Laboratory of Environment and Genes Related to Disease of Ministry of Education, Health Science Center, Xi'an Jiaotong University, Xi'an, China; ^2^Research Group of Cellular and Molecular Immunology, Vrije Universiteit Brussel, Brussels, Belgium; ^3^Laboratory of Myeloid Cell Immunology, VIB Center for Inflammation Research, Brussels, Belgium; ^4^Talent Highland, The First Affiliated Hospital, Xi'an Jiaotong University, Xi'an, China; ^5^In vivo Cellular and Molecular Imaging Laboratory, Vrije Universiteit Brussel, Brussels, Belgium

**Keywords:** Clec4F, nanobody, epitope binning, x-ray crystallography, Kupffer cells

## Abstract

Nanobodies that are derived from single-chain antibodies of camelids have served as powerful tools in diagnostics, therapeutics and investigation of membrane receptors' structure and function. In this study, we developed a series of nanobodies by a phage display screening building from lymphocytes isolated from an alpaca immunized with recombinant mouse Kupffer cell receptor Clec4F, which is involved in pathogen recognition by binding to galactose and N-acetylgalactosamine. Bio-panning selections retrieved 14 different nanobodies against Clec4F with an affinity ranging from 0.2 to 2 nM as determined by SPR. Those nanobodies mainly recognize 4 different epitopes as analyzed *via* competitive epitope binning. By analysis of the radioactivity in each organ after injection of ^99m^Tc labeled Clec4F nanobodies in naïve mice, we found that these nanobodies are targeting the liver. Furthermore, we performed a structural characterization at atomic resolution of two of the Clec4F nanobodies from different epitope groups, which revealed distinct features within the CDR2 and CDR3 regions. Taken together, we developed a series of nanobodies targeting multiple distinct recognition epitopes of the Kupffer cell-specific receptor Clec4F which may be useful for its structural and functional investigation as well as for use as molecular imaging and therapeutic agents.

## Introduction

Macrophages seem to adapt to different tissues with specific functions during development and adulthood, hence the tissue-resident macrophages are extremely heterogeneous (1). The identification of tissue-resident macrophages and their unique feature in relation to other immune cells has so far mainly relied upon the detection of their anatomical position (2, 3). Apart from the heterogeneity observed between different organs, even within the same tissues, there appear to be subpopulations of specialized macrophages that exhibit unique characteristics (4). Physically, Kupffer cells (KCs) are interspersed with fenestrated liver sinusoidal endothelial cells in a mosaic fashion to make up the sinusoidal lining. KCs express several cell-surface receptors and receptor complexes involved in immune stimulation (5). These include complement receptors (CRs), Fc-receptors, C type lectins (CLR), which are capable of directly binding microbial surface components such as sugars and polyanionic moieties, adhesion receptors, and receptors for polysaccharides of microbial and host origin (6–8). Targeting of markers expressed on KCs and inflamed epithelium offers perspectives for monitoring the inflammation development and understanding the type of specific cells and receptors involved (7). The most frequently used markers for KCs are CD68, CD11b, and F4/80 in mouse. The presence of F4/80 antigen (which becomes expressed as monocytes mature into tissue macrophages) on sinusoidal liver cells has been used to “define” KCs (9).

Recently, we have identified Clec4F as resident liver macrophage marker. Clec4F (C-type lectin domain family 4, member F), is a type II transmembrane glycoprotein member of the C-type lectin superfamily (10). Clec4F can bind galactose and N-acetylgalactosamine. The mouse Clec4F forms a trimeric coiled-coil interface within its heptad neck and constitutes conserved Ca^2+^-dependent carbohydrate-recognition domains, however as compared to other C-type lectin family members, Clec4F exhibits a distinct distance between the Ca^2+^ binding site within the trimer which is proposed to enable binding of specific glycans chain (11). Humans cannot encode a functional Clec4F receptor due to a mutation in the splice acceptor site of the last exon preventing appropriate splicing, and a missense mutation disrupting the sugar-binding site (12). Although Clec4F is present in rodents but not in humans, Clec4F is starting to be used as a marker in immunity and *in vivo* imaging in mouse disease models to study KCs^11^. Based on Clec4F, together with Tim4, Decisscher et al. were able to distinguish between monocyte-derived macrophages (MoMφs) and KCs in the Non-alcoholic steatohepatitis (NASH) liver (10, 13). Coupling of the diphtheria toxin receptor to the Clec4F promoter (KC-DTR mice) allowed the specific ablation of KCs and KC-DTR mice, providing a powerful tool for ZEB2 identification (14) as a KC-specific transcription factor. Clec4F-deficient mice produced far less cytokines than wild type littermates after intravenous injection of α-GalCer, which binds to CD1d and is a typical stimulator of active NKT cells (15), which suggests that Clec4F may be involved in presentation of glycolipid antigens or α-GalCer to NKT cells and thus could be an important ligand to activate liver KCs.

Molecular engineering of the heavy-chain antibodies' antigen-binding domains created the VHHs, having unique biophysical properties that render them attractive for biotechnological applications. The name nanobody (Nb) was coined to refer to the recombinantly-produced form of the VHH (16). As compared to other antibody fragments consisting of multiple domains, the Nb domains have a characteristic prolate ellipsoid shape that allows them to target cavities deep within their target antigens, therefore, Nb domains are frequently observed to target hidden or conformational target epitopes, which offers an added value for many applications (17). Owing to their unique properties, Nb domains have become increasingly popular for purposes of diagnostic or therapeutic applications in various disease areas, including infectious, inflammatory and oncologic diseases (18, 19).

Previously, we reported the construction of an immune phage display library for retrieving Clec4F nanobodies. Furthermore, a lead Clec4F nanobody, Nb 2.22, was identified to bind to KCs in FACS and in immunohistochemistry (20). It was also shown there that the ^99m^Tc-Nb 2.22 signal in liver upon *in vivo* imaging disappears after chlodronate liposome depletion of KCs, demonstrating that the *in vivo* signal in the liver is restricted to KCs (10). In the current manuscript, we provide more details on how we immunized an alpaca against Clec4F and isolated a number of Clec4F nanobodies that display high affinity. We demonstrated by epitope binning that these 25 Clec4F nanobodies mainly bind to 4 individual epitopes of Clec4F. The protein structure of Nb2.22 clearly reveals enhanced flexibility in the CDR3 as compared to Nb1.46. The series of nanobodies targeting multiple distinct epitopes of Kupffer cell receptor Clec4F may be beneficial for its structural and functional investigation as well as for future use in molecular imaging and therapeutic agents.

## Materials and Methods

### Production of Recombinant Mouse Clec4F

NS0-derived mouse CLEC4F/CLECSF13 protein, with an N-terminal 9-His tag was ordered from R&D Systems which the soluble extracellular domain Ala65-Gly548 of Clec4F was expressed in mouse myeloma cell line, and used for alpaca immunization, panning and ELISA screening, largely as described previously (21). VHH phage-display library was generated by RT-PCR on mRNA of peripheral blood lymphocytes isolated from an immunized alpaca (*Vicugna pacos*) and cloning in the vector pHen4. The repertoire of the VHH library was expressed on phages and panned against microplates coated with Clec4F antigen at a concentration of 10 μg/μl. After four rounds of selection against recombinant mouse Clec4F, individual colonies were picked from round 2 and round 3 of panning, and their VHH was expressed as soluble periplasmic protein.

### Enrichment Phage ELISA

Enrichment phage ELISA was performed for four rounds of bio-panning of phage-displayed Clec4F nanobodies. The antigen containing wells were coated with either 1 μg/ml mouse Clec4F protein, blank wells were coated with PBS. Output phages before panning (round 0) and after 4 rounds of bio-panning were used as ligands in an ELISA. Color was developed using mouse anti-M13 antibody followed by anti-mouse AP and AP substrate. The recombinant VHH containing periplasm was extracted and was tested for antigen recognition in phage enrichment ELISA and the clones which generated a positive signal on Clec4F protein (OD450 nm signal of each clone was divided by signal of well without antigen and considered positive if the resulting ratio was ≥3) were chosen for DNA sequencing.

### Nanobody Production and Purification

The Clec4F-specific nanobody gene sequences were inserted into the pHEN6c plasmid and transfected into E. coli WK6 host cells. Hexahistidine-tagged (His-tag) Clec4F nanobodies were extracted from E. coli WK6 periplasmic space by osmotic shock. All the extracted proteins were expressed in 400 mL medium and purified by immobilized metal affinity chromatography (IMAC) using the Ni-NTA column and the buffer was exchanged from 0.5 M imidazole elution buffer to PBS by dialysis. The purity and size of the nanobody was detected by SDS-PAGE and Coomassie brilliant blue staining.

### Affinity Evaluation and Epitope Mapping of Clec4F Nanobodies

After purification, the concentration of Clec4F nanobodies was determined by the Nanodrop spectrophotometer using the theoretical extinction coefficient of the nanobody calculated from its amino acid sequence (7). All selected nanobodies bound to Clec4F antigen with high affinities in nM range as determined by SPR. Nb affinity experiments were carried out by surface plasmon resonance (SPR, Biacore T200, GE Healthcare). The Clec4F nanobodies were flown at 30 μl/min, at different concentrations (1–400 nM) over 400 RU (resonance units) of recombinant mouse Clec4F protein coupled to a CM5 chip. The affinity constants were determined using a 1:1 binding model using the BIACORE evaluation T200 software (GE Healthcare).

Epitope mapping of Clec4F nanobodies was also investigated by SPR. Because Nb 1.46, 12.75, and 5.10 were very difficult to elute and regenerate from the Clec4F coated CM5 chip, we excluded them from the epitope binning. Briefly, after a 1st Nb was injected and reached its saturation plateau, a mix of the 1st Nb and a 2nd Nb was injected to evaluate whether the 2nd Nb caused extra binding RU. Next, the sensorgram for each Nb pair was drawn to investigate the competition profiles.

### ELISA

Clec4F (1 μg/mL) was coated directly on 96-well plates, overnight at 4°C. Free protein binding sites were blocked by 4% skimmed milk in PBS for 2 h at room temperature. Next, a series dilution of Clec4F nanobodies in 100 μL 1% skimmed milk PBST were added. Detection of antigen bound Nb was performed by anti-His-tag and subsequently by anti-mouse IgG- alkaline phosphatase antibody. The OD405 nm values of each well were subtracted with the blank (signal from well without Clec4F).

### ^99m^Tc-Nanobody Labeling and Pinhole SPECT/μCT Analysis

Based on the yield and purity of the nanobodies, one Nb representative for each group was selected for further analysis. Nanobodies were labeled with ^99m^Technetium (^99m^Tc) *via* their His-tag through tricarbonyl chemistry as described before (22, 23). Hereby, the (^99m^Tc) tricarbonyl precursor was prepared in accordance to the IsoLinkTM kit (Covidien, St. Louis, USA). The kit reduces and carbonylates [^99m^Tc]TcO_4_ into ([^99m^Tc](CO)_3_(H_2_O)_3_)^+^ after heating (100°C for 20 min). This precursor (500 μl) was next incubated at 50°C for 1 h with 50 μg of the His-tagged Nb. The [^99m^Tc] tricarbonyl precursor forms a tridentate coordinated complex with every other histidine residue in the hexahistidine complex. The ^99m^Tc-labeled Nb (^99m^Tc-Nb) solution was subsequently purified on a NAP-5 column (GE Healthcare), and passed through a Millex-GV4 0.22-mm filter (Millipore). After ^99m^Tc-radiolabeling and subsequent purification steps, labeling efficiencies, reflecting the amount of the added ^99m^Tc that ended up coupled to the filtered nanobodies, ranged between 50 and 70% for the various nanobody preparations. Radiochemical purities, reflecting non-free radioactivity coupled to the nanobody in the final filtered preparation, were determined by instant thin-layer chromatography using acetone as mobile phase and were at least 99% for all nanobodies. Mice were injected intravenously with 80–100 μl of ^99m^Tc-Nbs, corresponding to 52.79 ± 19.63 MBq (3 mice ± SEM) per mouse. At 1 h post-injection, mice were sacrificed, tissues were dissected and weighed, and their radioactivity content was measured using an automated γ-counter (Cobra II Inspector 5003; Canberra-Packard, Schwadorf, Austria). Organ uptake was calculated as the percentage of injected radioactivity per gram (%IA/g) and corrected for decay (23). All applicable institutional and/or national guidelines for the care and use of animals were followed and all animal experiments were approved by the Ethical Committee for Animal Experiments of the Vrije Universiteit Brussel (Lab Accreditation Number: LA1210220).

### Crystallization and Data Collection

All crystallization trials were carried out with the sitting-drop vapor-diffusion approach at room temperature. Clec4F nanobodies were purified by size exclusion chromatography on columns pre-equilibrated with 20 mM Tris pH 7.5, 150 mM NaCl and 5% Glycerol. The fractions containing the nanobodies were pooled and concentrated to around 15 mg/mL. The crystallization trials were further carried out by mixing 0.5 μL protein complex with an equal volume of reservoir solution. The Nb2.46 was crystallized in 0.02 M Magnesium chloride hexahydrate, 0.1 M HEPES, pH 7.5, 22% poly acrylic acid sodium salt 5100 and the Nb2.22 crystals were observed in 0.05 M Citric acid, 0.04 M BIS-TRIS propane pH 5.0, 16% polyethylene glycol 3,350 respectively. All the crystals were harvested from the crystallization drop and flash frozen with a cryostream. Single crystals data were collected in the Shanghai Synchrotron Radiation Facility (SSRF) BL18U1 and BL19U1 at a wavelength of 0.98 Å at 100 K. The Nb1.46 crystal diffracted to 1.98 Å with α = β = γ = 90°, a = b = 73.5 Å, c = 94.8 Å in the P41212 space group, and the Nb2.22 crystal diffracted to 2.70 Å with α = β = γ = 90°, a = b = 80.5 Å, c = 107.7 Å, in the P42 space group.

### Structure Determination

Data were processed with XDS package (24). The structures of Clec4F nanobodies were determined *via* molecular replacement using Phaser implemented in the Phenix package using nanobody from 5IMM as search model (25). The model was manually improved with the COOT program (26) and refinement was further done using Phenix (25). The interaction interface was calculated by PDB PISA. Figures were generated from PyMOL program. Data collection and refinement statistics are summarized in Supplementary Table 1. Crystallographic coordinates and structure factors were deposited to the Protein Data Bank with access codes 7DJX and 7DJY for Nb1.46 and Nb2.22.

### Statistical Analysis

Statistical analyses were conducted using the one-way ANOVA assuming unequal variances. Prism 6.0 (GraphPad software) was used for statistical analyses and graph creation. *P*-values ≤ 0.001 were considered significant.

## Results

### Mouse Clec4F Nanobody Generation

**Figure 1:**
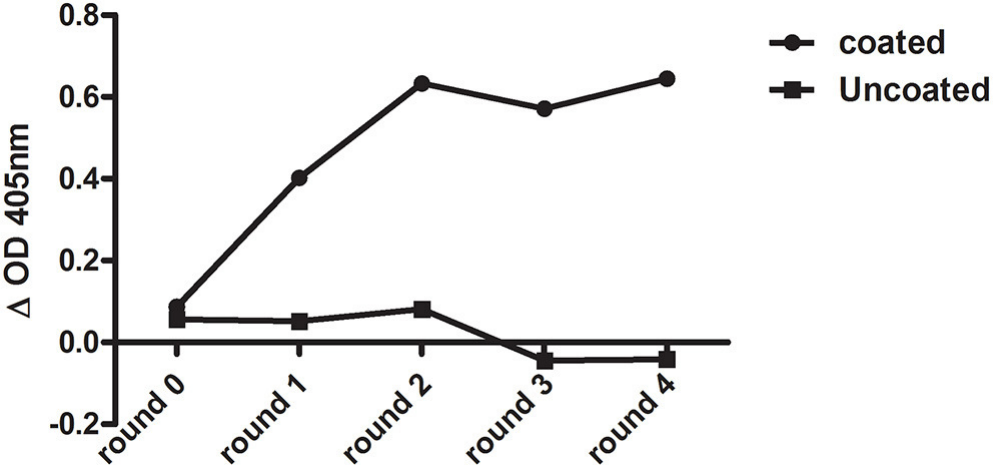
Enrichment of Clec4F-specific nanobody after each round of bio-panning as measured by phage ELISA. Wells were coated with either 1 μg/ml mouse Clec4F recombinant protein (coated), or only with PBS (uncoated). Output phages before panning (round 0) and after 4 rounds of biopanning were used as ligands in an ELISA.

After six rounds of immunization with Clec4F recombinant protein, total mRNA from peripheral blood lymphocyte cells (PBLs) was isolated, then the cDNA was generated using RT-PCR. The VHH library with a size of 7.27 × 10^7^ individual clones was expressed on M13 phages and panned against recombinant Clec4F protein coated in microplate wells. In total, four rounds of phage panning were performed. After the third round of panning, antigen-recognizing phages were enriched by a factor of 4.4 × 10^4^. The antigen-specific phages were confirmed in a phage enrichment ELISA against micro-plate coated Clec4F (Figure 1). Periplasmic extracts were generated of 94 colonies from the 2nd and 47 colonies from the 3rd round of panning, respectively. When these extracts were tested in ELISA against recombinant mouse Clec4F protein, based on the specific signal vs. background signal ratio, 102 out of the 141 Nb clones demonstrated specific antigen binding and were sequenced (Table 1).

**Table 1:** PE-ELISA of individual antigen-binding Clec4F nanobodies from panning of round 2 and 3.

**Specific signal vs. background signal**	**Round 2**	**Round 3**	**Total**
>3-fold	69	33	102
2–3-fold	7	6	13
<2-fold	18	8	26
Total colonies	94	47	141

*The number of colonies are shown with respect to the specific signal generated during the assay. Colonies with the best signal strength difference vs. background signals were DNA sequenced*.

**Figure 2:**
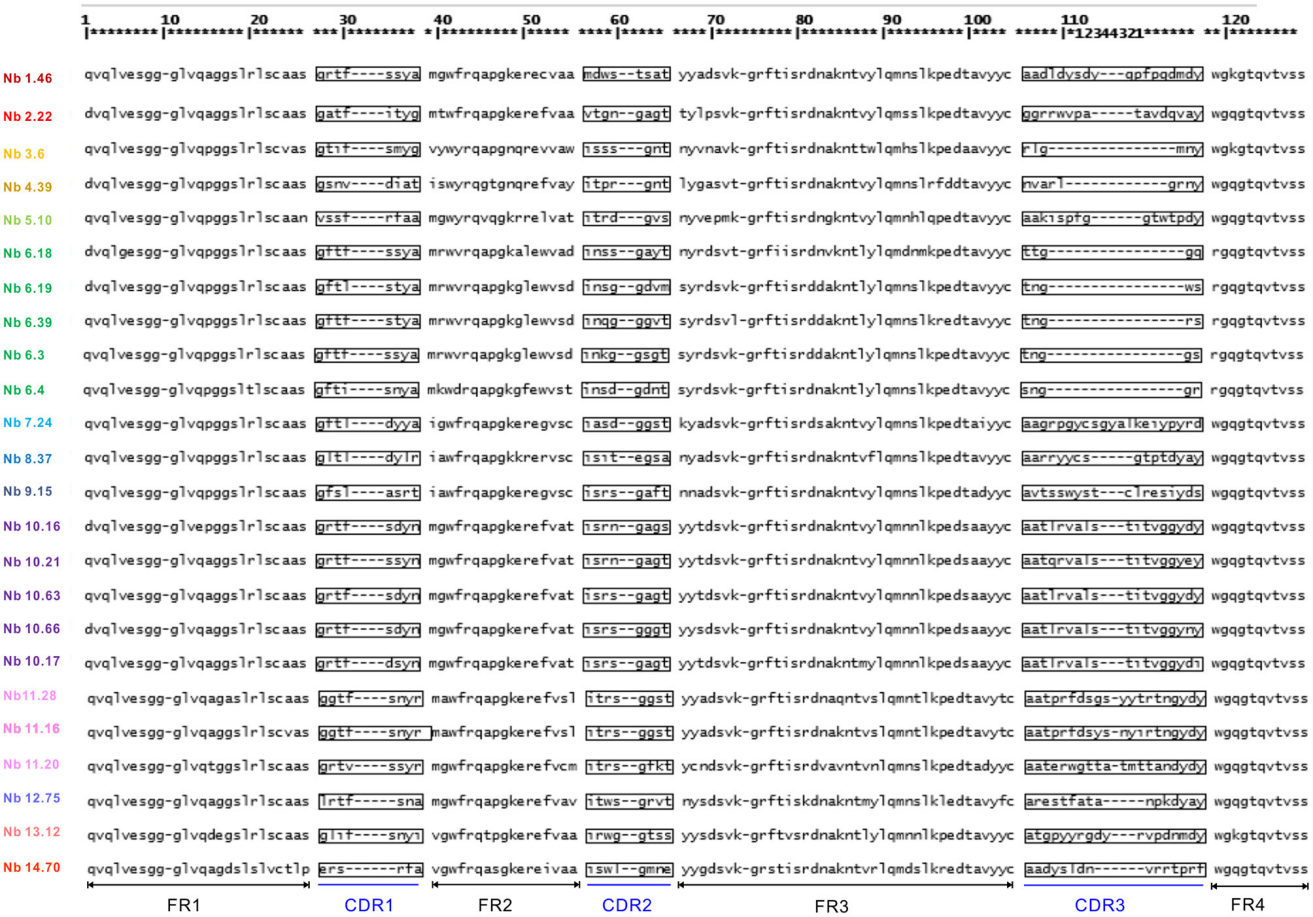
Amino acid sequence of Clec4F nanobodies (numbering according to IMGT). Deduced amino-acid sequences of different nanobodies, consisting of complementarities determining regions (CDR) sequences alternated with structural framework region (FR) sequences.

Within the VHH, there are three complementarity determining regions (CDRs) or antigen-binding loops. The 3 CDR regions define the unique binding specificity to the antigen epitope, whereby the length and sequence variability of these loops determine the antigen binding affinity. The CDR regions are separated by relatively invariant regions to support the CDRs, called framework regions. Among 102 positive clones, 24 distinct VHH fingerprints were identified according to the IMGT unique numbering system, belonging to 13 different groups based on their unique CDR3 regions. The other groups not only showed a unique CDR3 but also unique CDR1 and CDR2 sequences. Representative clones were selected from each group, differing in a few framework region amino acids (Figure 2). In total 23 nanobodies were successfully recloned to the pHEN6c vector for expression with a his-tag at the carboxy-terminus.

### Production and Purification of mClec4F Nanobodies

WK6 *E. coli* cells were transformed with the plasmid constructs after which the periplasmic expression of Clec4F nanobodies was performed. All the nanobodies were produced in 400 ml as an initial trial and purified by IMAC and buffer exchanged by dialysis (Supplementary Table 2). The yield of nanobodies varied from 1.1 mg to 16 mg per liter. Nanobodies Nb7.24, Nb4.39, Nb11.63, and Nb6.39 were expressing poorly, therefore we excluded them from later experiments.

**Figure 3:**
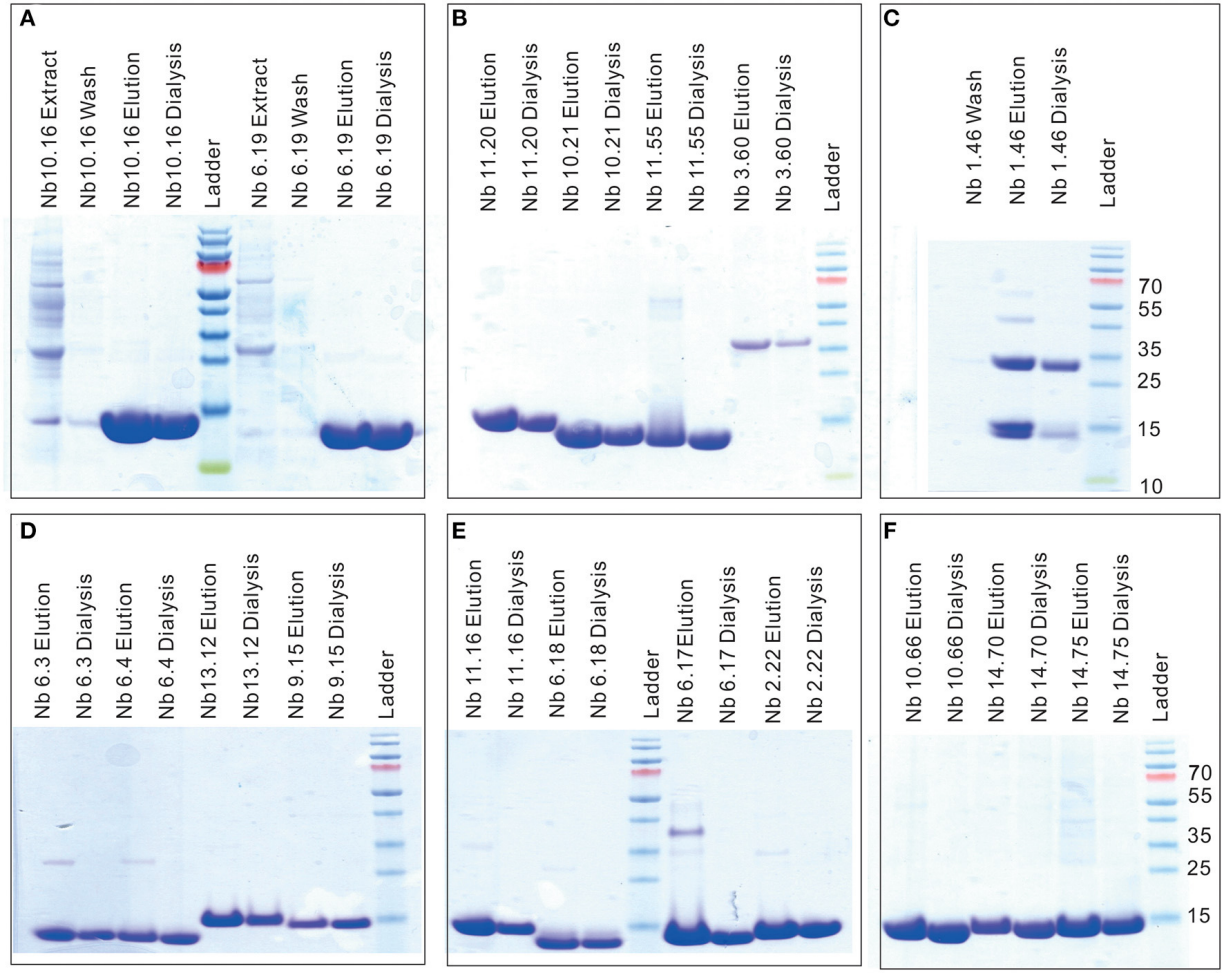
SDS-PAGE to confirm the purity and size of Clec4F nanobodies. Ten percentage SDS-PAGE revealed the presence of **(A)** Nb10.16, Nb6.19 **(B)** Nb11.20, Nb10.21, Nb11.55, Nb3.60 **(C)** Nb1.46 **(D)** Nb6.3, Nb6.4, Nb13.12, Nb9.15 **(E)** Nb11.16, Nb6.18, Nb6.17, Nb2.22 **(F)** Nb10.66, Nb14.70 and Nb14.75, in (periplasmic) extract, wash flow through (10 mM imidazole-PBS), elution buffer (500 mM imidazole-PBS) and Nb after dialysis (PBS). It showed high purity and a characteristic Nb size of around 14 kD; Nb1.46 and Nb3.60 show dimerized material. M lane: relative molecular mass marker (kDa).

The SDS-PAGE of nanobodies Nb10.16, Nb6.19, Nb11.20, Nb10.21, Nb11.55, Nb10.66, Nb14.70, Nb12.75, Nb6.3, Nb6.4, Nb13.12, Nb9.15, Nb11.16, Nb10.17, Nb6.18, Nb2.22, Nb8.37 in elution buffer after IMAC purification and dialysis showed high purity and the characteristic size of nanobodies around 14 kD (Figure 3). The preparations of nanobodies Nb1.46 and Nb3.60 revealed presence of dimers (Figures 3C,D). This was expected for Nb1.46 as it contained three cysteines, two of which form the conserved disulphide bond and an extra cysteine at position 52 (IMGT numbering) that could form an interdomain disulfide bond. The dimerization of Nb3.60 was unexpected and might be provoked by the short CDR3 whereby the FR4 of two nanobodies are swapped during folding.

### The Binding Capacity of the Nanobodies to Clec4F Determined by Surface Plasmon Resonance

Binding kinetics of nanobodies targeting Clec4F were determined using surface plasmon resonance (SPR). The kinetic interaction between Clec4F nanobodies and Clec4F yielded a picomolar equilibrium dissociation constant (K_D_) for most nanobodies. The comparison of the *k*_*on*_*, k*_*off*_, and K_D_ values of Clec4F nanobodies revealed that 12 nanobodies were found to display excellent off-rates from Clec4F (Table 2). The corresponding *k*_*off*_ values of nanobodies from group 2, 6, 10, and 11 were superior compared to the others. Furthermore, the difficult elution and regeneration of Nb 1.46, 12.75, and 5.10 from the Clec4F conjugated SRP chip may also suggest they have a good affinity. For the dimerized Nb1.46 this suggested that at least one entity was active in antigen binding.

**Table 2:** Kinetic rate and equilibrium binding constants of the Clec4F nanobodies, measured using surface plasmon resonance on immobilized recombinant mouse Clec4F protein.

**No**	**Name**	**Round**	**Group**	**k_**on**_ (M^**−1**^s^**−1**^)**	**k_**off**_ (s^**−1**^)**	**K_**D**_ (M)**	**nM**
1	Nb1.46	R2-46	G1	The regeneration is not feasible, not measured.			
2	Nb2.22	R3-22	G2	8.242E + 6	0.002213	2.685E-10	0.26
3	Nb3.60	R2-60	G3	7.687E + 6	0.004750	6.180E-10	0.61
4	Nb4.39	R2-39	G4	Poorly expressed.			
5	Nb5.10	R3-10	G5	The regeneration is not feasible, not measured.			
6	Nb6.3	R3-3	G6	2.868E + 6	8.484E-4	2.958E-10	0.29
7	Nb6.4	R3-4	G6	1.155E + 6	0.002314	2.003E-9	2.00
8	Nb6.18	R3-18	G6	1.482E + 6	7.559E-4	5.1001E-10	0.51
9	Nb6.19	R2-19	G6	1.302E + 6	6.125E-4	4.704E-10	0.47
10	Nb6.39	R3-39	G6	Poorly expressed.			
11	Nb7.24	R2-24	G7	Poorly expressed.			
12	Nb8.37	R3-37	G8	1.573E + 6	0.001243	7.902E-10	0.79
13	Nb9.15	R3-15	G9	1.189E + 6	0.03171	2.668E-8	26
14	Nb10.16	R2-16	G10	1.576E + 6	8.252E-4	5.237E-10	0.52
15	Nb10.17	R3-17	G10	9.082E + 6	0.001584	1.744E-10	0.17
16	Nb10.21	R2-21	G10	4.619E + 6	0.002233	4.835E-10	0.48
17	Nb10.66	R2-66	G10	4.280E + 6	0.001345	3.143E-10	0.31
18	Nb11.16	R3-16	G11	2.320E + 7	0.003921	1.690E-10	0.16
19	Nb11.20	R2-20	G11	3.459E + 6	0.001395	4.033E-10	0.40
20	Nb11.28	R3-28	G11	5.432E + 6	0.006535	1.203E-9	1.20
21	Nb11.55	R2-55	G11	4.332E + 6	0.002150	4.964E-10	0.49
22	Nb11.63	R2-63	G11	Poorly expressed.			
23	Nb12.75	R2-75	G12	The regeneration is not feasible, not measured.			
24	Nb13.12	R3-12	G13	2.788E + 6	0.003097	1.111E-9	1.11
25	Nb14.70	R2-70	G14	3.061E + 7	0.06062	1.980E-9	0.19

*The affinities (K_D_), on-rate (k_on_), off-rate (k_off_) are calculated based on fitting of observed interaction curves of a dilution series of nanobodies*.

### Clec4F Nanobodies' Affinity and Epitope Binning

**Figure 4:**
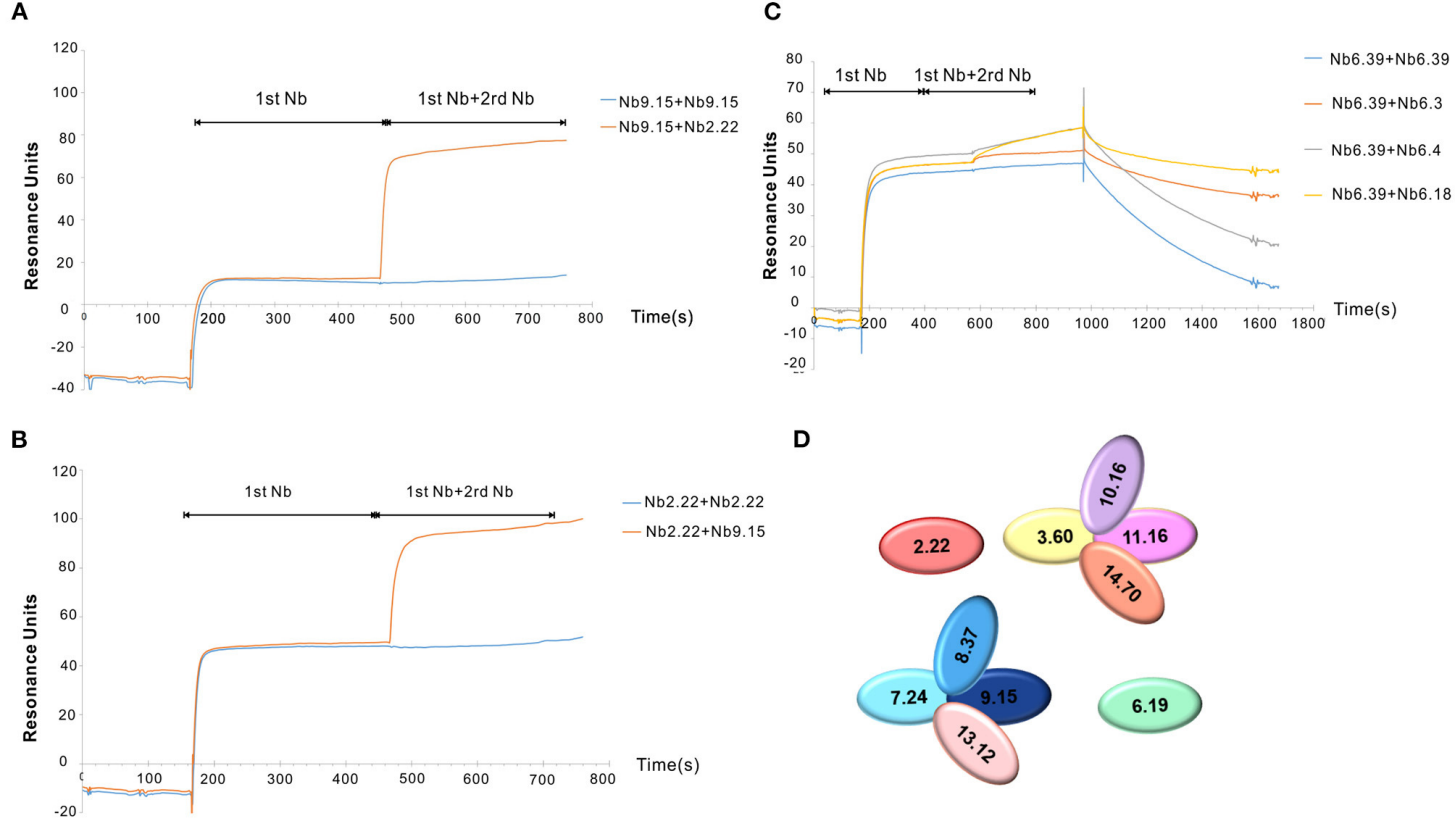
Clec4F nanobodies epitope binning by SPR. Examples of lack of overlap between Nb2.22 (2rd Nb) and Nb9.15(1st Nb) epitopes on Clec4F antigen **(A)** and vice versa of Nb9.15 (2st Nb) and Nb2.22 (1st Nb) to Clec4F **(B)**, indicating that these bind to different epitopes. **(C)** The inhibitory effect of nanobodies within group 6. RU refers to resonance units. **(D)** Summary of Clec4F Nb epitope mappings with different colors indicating different epitope groups. The nanobodies are named according to different groups in CDR3.

Clec4F nanobodies from groups 2, 3, 6, 7, 8, 9, 10, 11, 13, and 14 were selected for epitope binning using SPR. Since it was very difficult to completely remove Nb1.46, Nb5.10, and Nb12.75 after the regeneration step, these nanobodies were excluded from the epitope binning experiment. The Clec4F protein was immobilized on a chip and competitive binding between the nanobodies was analyzed by running the nanobodies subsequently over the chip. The first Nb was flown over the sensor using a concentration of 50 nM, which is 10–20-fold of the Nb KD to saturate its epitope. After the 1st Nb binding reached equilibrium, a mix of the 1st Nb and a 2nd Nb was injected to evaluate whether this 2nd Nb caused extra binding resonance units (RU), indicating that these nanobodies bind to independent epitopes on Clec4F. As an example, the Nb9.15 and Nb2.22 did not affect each other's binding, which demonstrated that they recognize different epitopes since RU levels doubled upon binding of the second nanobody (Figures 4A,B). Next, all the possible combinations of Nb pairs within the different groups were tested. For example, Nb6.3, Nb6.4, or Nb6.18 did not cause an extra RU upon the binding of Nb6.39, indicating that all members from group 6 bind to the same epitope (Figure 4C). These results corresponded with the similar sequence alignment in CDR3. Finally, the interaction studies between the representative nanobodies from each group are summarized in Supplementary Table 3. Briefly, the SPR study revealed that 4 different epitope groups existed among Nb sequence groups 2, 3, 6, 7, 8, 9, 10, 11, 13, 14, which are graphically depicted in Figure 4D.

### *In vivo* Biodistribution of Clec4F Nanobodies

**Figure 5:**
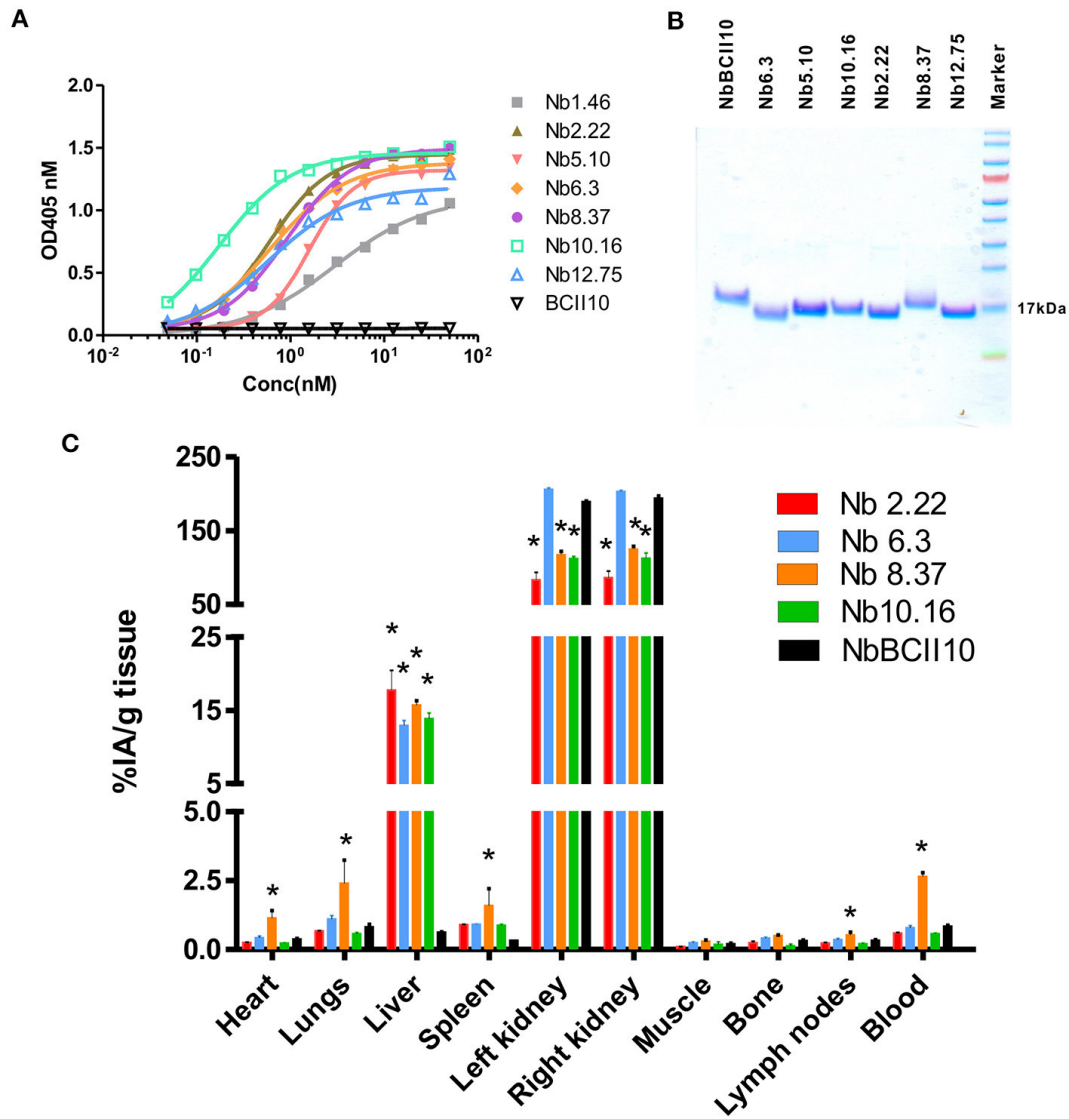
Binding characteristics of Clec4F nanobodies. **(A)** Binding of increasing concentrations of Clec4F nanobodies was tested on recombinant Clec4F protein using ELISA. **(B)** SDS-PAGE confirmed the purity and size of selected Clec4F nanobodies after purification by size exclusion chromatography. **(C)** Biodistribution of radiolabeled Clec4F Nanobodies in naive mice. Four distinct ^99m^Tc-Clec4F nanobodies and ^99m^Tc-NbBCII10 control Nb were injected in naive mice (mean of 3 mice ± SEM). Radioactive content of each organ was measured in a γ-counter and expressed for each Nb as a percentage of injected radioactivity per gram of tissue (%IA/g), *P < 0.001 vs. ^99m^Tc-NbBCII10 control Nb.

Clec4F nanobodies that bind non-competitively to Clec4F were purified by size exclusion chromatography and retested for affinity and purity (Figures 5A,B). Four selected nanobodies (Nb3.3, Nb10.16, Nb2.22, and Nb8.37) which exhibited the highest affinity within their epitope group were labeled with ^99m^Technetium (^99m^Tc) *via* their his-tag through tricarbonyl chemistry, purified and filtered. After ^99m^Tc-radiolabeling and subsequent purification steps, labeling efficiencies, reflecting the amount of the added ^99m^Tc that ended up coupled to the filtered nanobodies, ranged between 50 and 70% for the various nanobody preparations. Radiochemical purities, reflecting non-free radioactivity coupled to the nanobody in the final preparation, were at least 99% for all nanobodies. These radiolabeled nanobodies were injected intravenously, followed by dissection of the different organs and measurement of the radioactivity in each organ, to test whether these ^99m^Tc-Nbs could target Clec4F positive tissue (liver) in the naive mouse *in vivo*. The non-targeting control NbBCII10 showed only a significant signal in the kidneys, reflecting Nb retention in the kidneys after filtration. With the exception of Nb8.37, which showed a significant radioactive signal in blood and other tissues, the other Clec4F nanobodies showed a similar biodistribution pattern: specific uptake was detected in the liver, but not in any of the other organs (Figure 5C). ^99m^Tc-Nb2.22 with a relative high *in vitro* affinity for the recombinant target (0.3 nM) also demonstrated superior accumulation in the liver (*p* < 0.05). So, based on these biochemical properties and specifically on the liver targeting properties, Nb2.22 targeting a unique epitope was selected as lead Clec4F nanobody for future KC imaging studies.

### Structural Characterization of Nb1.46 and Nb2.22

**Figure 6:**
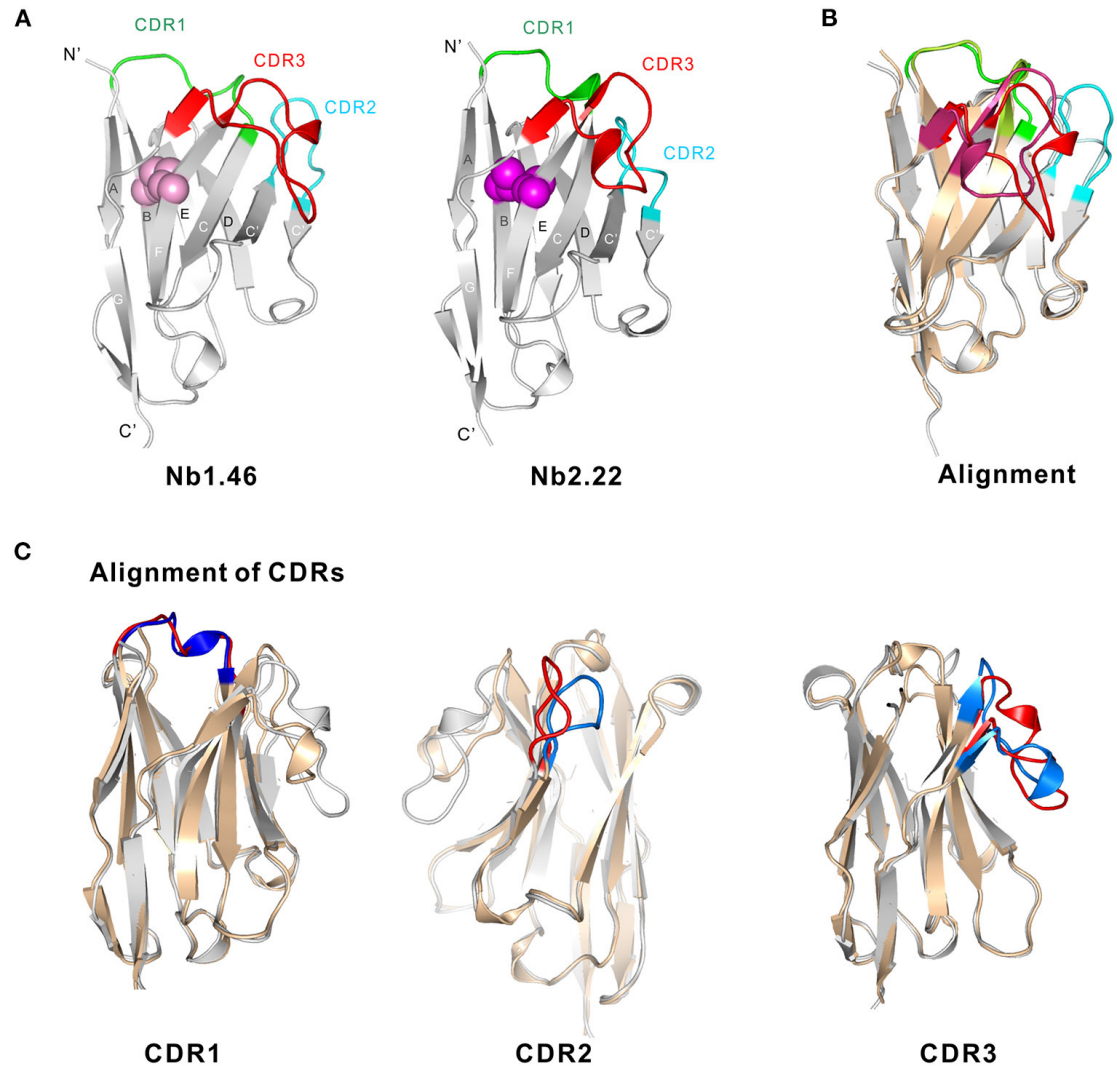
Structural basis of Nb1.46 and Nb2.22. **(A)** The cartoon view of the Nb1.46 and Nb2.22, the CDR1, CDR2, CDR3 are shown in the color green, cyan and red, respectively, the cysteine involved in disulfide bond formation are denoted as pink sphere for Nb1.46 and violet sphere for Nb2.22. **(B)** The alignment of Nb1.46 to Nb2.22, whereby Nb1.46 is colored in gray and Nb2.22 is colored in wheat. **(C)** The details of CDRs alignment of Nb1.46 to Nb2.22, whereby Nb1.46 is colored in gray and its CDR in red, whereas Nb2.22 is colored in wheat and its CDR in blue.

To investigate the diversity of the nanobodies' epitopes and to allow to in the future further optimize them based on rational design, we determined the crystallographic structure of these nanobodies. We tried to obtain crystals from several of the anti-Clec4F nanobodies. Unfortunately, we could only obtain crystals for Nb1.46 and Nb 2.22. Nb1.46 has an unpaired cysteine in FR2, and a lysine in FR4 which is unusual for nanobodies. It may explain that Nb1.46 forms dimers (Figure 3) and has a lower affinity as compared to Nb2.22, as detected by ELISA (Figure 5A). The Nb1.46 and Nb2.22 structures were determined through molecular replacement using a Nb119, which is a Nb with specificity against Vsig4, another KC marker, as a template to a resolution of 1.98 and 2.70, respectively (27). The overall structure of Nb1.46 and Nb2.22 contains 10 β-strands as the canonical immunoglobulin fold of VHH in two antiparallel β-sheets. The folded VHH fragment forms an extended interface that interacts with the antigen, known as paratope. The Nb1.46 has CDR1 to 3 comprising 8 residues (Glu^27^ to Ala^38^), 8 residues (Met^51^ to Thr^58^) and 16 residues (Asp^99^ to Tyr^114^), respectively. The Nb2.22 has CDR1 to 3 comprising 8 residues (Glu^27^ to Tyr^38^), 8 residues (Val^51^ to Thr^58^) and 14 residues (Arg^99^ to Tyr^112^), respectively (Figure 6A). Nb1.46 and Nb2.22 have 8 residues in CDR1 and among them there are four residues that are different.

As shown in the structure alignment, the CDR1 of Nb1.46 and Nb2.22 almost coincide (Figure 6B). However, Nb1.46 and Nb2.22 have 8 completely different residues in the CDR2, resulting in the CDR2 loop bending into different orientations since CDR2 of Nb2.22 has residues Gly^58^ and Gly^62^ that make the surface more hydrophobic. Antigen binding is mediated by non-covalent interactions, mainly involving amino acids in the CDRs, mostly in CDR3. Interestingly, the CDR3 of Clec4F nanobodies includes a short β-strand. Nb2.22 has a shorter version of the CDR3 loop as compared to Nb1.46 indicating a completely different pattern of binding with Clec4F. Nb1.46 and Nb2.22 also harbor two cysteines (Cys^23^ and Cys^104^) that form the disulfide bridge connecting the two β-strands to constrain the antigen-binding loops and maintain the flexibility (Figure 6C). However, an interdomain disulphide bond of Cys^52^ may mediate the dimer formation in the Nb1.46.

## Discussion

The liver performs a wide range of functions, including detoxification, protein synthesis, production of biochemicals necessary for digestion, clearance of macromolecules from the blood and their metabolization (28). The liver KCs play an important role in surveillance, uptake of immune complexes, tumor cells, liposomes, lipid microspheres, iron, immune complexes and other blood-borne particulates and as regulatory and effector cells in immune responses to infectious agents and other conditions challenging liver homeostasis. To optimally dissect the role of the various cell types in the liver, it is a crucial asset to identify the resident KCs from other myeloid cells such as circulating monocytes and different types of DCs or recently immigrated macrophages within populations of liver cells after isolation and under inflammatory conditions.

Clec4F has a carbohydrate-recognition domain (CRD) that recognizes glycans in a Ca^2+^-dependent (C-type) manner, an N-terminal cytoplasmic signaling domain, a transmembrane hydrophobic helix, and a heptad neck region, which stabilizes trimer formation. As we have shown in our previous studies, Clec4F is only expressed by Kupffer cells and is absent in infiltrating monocytes. Therefore, Clec4F can be used as a specific Kupffer cell marker to study the roles of distinct populations present in the liver (29). The loss of Clec4F^+^ Kupffer cells was shown to be a sign of extensive liver damage upon Crimean-Congo hemorrhagic fever virus (CCHFV) infections in mice (30). Clec4F has also been shown to be necessary for the clearance of desialylated platelets due to the recognition of exposed galactose epitopes (5). Moreover, Clec4F was reported to be involved in the *Listeria monocytogenes* infection in mouse liver (9).

A few years ago, we have reported that molecular imaging with Kupffer cell-targeting nanobodies can be used for diagnosis and prognosis in mouse models of liver pathogenesis, whereby we used nanobodies targeting two distinct receptors on Kupffer cells, namely Vsig4 and Clec4F (20). The nanobodies against these two targets were used as tools for imaging therein, but their generation was not the focus of that publication. Meanwhile, we have also reported the structure of a truncated trimeric mouse Clec4F containing the CRD and part of the heptad neck domain. The Clec4F trimeric structure reveals two unique conserved calcium-binding sites, which differ from that of Langerin and other C-type lectins, which may contribute to the unique recognition pattern of Clec4F (11). Having solved the structure of Clec4F, has renewed the interest in the anti-Clec4F nanobodies and we have now also solved the structure of these anti-Clec4F nanobodies. In the current manuscript, we provide more detailed information on the initial generation and characterization of the nanobodies, including nanobody sequence data, epitope binning data and initial *in vivo* biodistribution data of various Clec4F nanobodies, which were not published before, and have combined these with our new findings on the crystal structure of Nb1.46 and Nb2.22.

Clec4F-specific nanobodies were obtained by direct cloning of the VHH genes from B-cells obtained from an immunized alpaca, and selection *via* phage-display and bio-panning. The Clec4F nanobodies showed good diversity, as reflected in the number of different sequences obtained after two and three rounds of panning. Twenty-five nanobodies were categorized into 14 groups based on sequence identity in their CDR3, which is the region of the VHH with the largest variability. In these 25 Clec4F nanobodies the length of CDR3 is in the range 6-21AA, whereby the nanobodies from group 6 have the shortest CDR3. Within each group these nanobodies have one or more mutations in other regions such as CDR1 and CDR2, which may also be related to their differences in binding capacity and expression levels. These 25 nanobodies were expressed in *E. coli* and purified from 400 mL cultures in TB medium, with variable yields. These nanobodies showed a limited variation in molecular weight, as shown by using SDS PAGE analysis. The K_D_ values of these nanobodies are in the range from 0.19 to 26 nM. Using a competitive assay *via* SPR we could determine that Nb 2.22 from Group 2 and Nb 6.19 from Group 6 bind to two different epitopes of Clec4F. The nanobodies of group 3, 10, 11, and 14 seem to inhibit each other's binding, suggesting they bind to a common epitope. The nanobodies of group 7, 8, 9 and 13 form a fourth epitope group. Therefore, we can distinguish at least four main epitope groups among these mouse Clec4F-binding nanobodies. Based on *in vivo* liver biodistribution in naive mice, we have observed that Nb 2.22, 3.3, 8.37, and Nb10.16 effectively target the liver. Of note, additional validation data of the Nb2.22, which was selected as lead Clec4F nanobody for KC imaging studies, have been published before (20). In particular, we have shown using Clec4F-DTR depleter mice, allowing for the specific depletion of KCs when treated with DT, that accumulation of the ^99m^Tc-labeled Nb2.22 is not observed in the liver of KC-depleted mice. Similarly, the liver accumulation of ^99m^Tc-Nb 2.22 also disappears in liver after chlodronate liposome depletion of phagocytes. These data confirm that the *in vivo* imaging signal in the liver is restricted to KCs. Moreover, immunofluorescence microscopy using Nb 2.22 on liver sections of naive mice has revealed that Clec4F co-stains F4/80-expressing cells. Finally, in flow cytometry analysis on liver single cell suspensions, Nb 2.22 stains CD11b^int^ F4/80^+^ KCs, but not F480^low^ CD11b^high^ inflammatory monocytes or Ly6G-expressing polymorphonuclear cells (20).

To have a basis for better understanding of the mode of action of these nanobodies and the different affinities, the crystal structures of Nb1.46 and Nb2.22 were obtained. The Nb1.46 has an unpaired cysteine in the FR2. Nb2.22 has a conserved intra-domain disulfide bond giving rise to a disulfide bridge joining the CDR1 and CDR3, as is often found in other conventional VHH domains. Such bridge would turn the loop into a more rigid structure, a relevant feature when considering its thermodynamic consequences. Furthermore, the polar amino acid substitutions of Glu^49^ and Arg^50^ increase the hydrophilicity of the VHH surface. Also residues Gly^58^ and Gly^62^ in CDR2 make the surface more hydrophobic, resulting in the CDR2 loop bending into a different orientation as compared to that one of Nb1.46. We also notice that the substitutions at positions Phe^42^ and Cys^52^ (Nb1.46) or Phe^52^ (Nb2.22) cause a net shift of the bulky hydrophobic groups toward the center of the sheet and the CDR3 loop folds over to these residues to make them solvent inaccessible. CDR3 of Clec4F nanobodies form a large protruding loop and together with CDR1 and CDR2, the antigen binding surface is as large as that of a scFv. Therefore, the antigen binding capacity of VHH and scFv is often in the same range.

Clec4F is a potential candidate biomarker restricted to the liver microenvironment. In the present study, we generated and evaluated anti-Clec4F nanobodies which are small binding moieties, that provide high affinity, but smaller size as compared to complete antibodies. These nanobodies lack the Fc region, and are therefore unable to recruit Fc-mediated effector activity. The superior penetration potential of nanobodies due to their smaller size, combined with high affinity target binding and fast clearance from the circulation, represents an ideal basis for imaging purposes (21, 31). By blocking the targets, nanobodies have been reported as efficient enzyme inhibitors for multiple usages (32) such as inhibiting the HIV-1 replication by blocking the Rev protein as an intrabody (33). The ability of nanobodies to recognize different antigenic regions on a protein such as cryptic, hidden or conformational target epitopes offers an added value for their applications. Finally, nanobodies can be easily expressed in *E. coli* where they can be economically produced as soluble and non-aggregating recombinant proteins in high yields (34).

In conclusion, our study has revealed a group of nanobodies that recognize distinct epitopes on Clec4F. Two of the Clec4F nanobodies were crystallized and the structures were determined through molecular replacement using a Nb119 as a template to a resolution of 1.98 and 2.70, respectively. These two nanobodies share many structural features, but the Nb1.46 and Nb2.22 CDR2 loops bend into different orientations, related to the 8 completely different residues in the CDR2. The Clec4F nanobodies presented here enable further investigation of Clec4F biological function using antibody-related methods such as flow cytometry, immunostaining, antigen purification and molecular imaging, as well as offer potential for developing specific isolation of KCs *in vitro* by Nb based immune affinity methods. Future work on these nanobodies will address their galactose neutralizing capacity on Clec4F. The determined molecular structure presented here will help in developing blocking nanobodies, which may find applications in blocking Clec4F interactions in disease models such as *Listeria monocytogenes* infection.

## Data Availability Statement

The datasets generated in this study can be found in online repositories. The names of the repository/repositories and accession number(s) can be found below: http://www.wwpdb.org/, 7DJX; http://www.wwpdb.org/, 7DJY.

## Ethics Statement

All applicable institutional and/or national guidelines for the care and use of animals were followed and all animal experiments were approved by the Ethical Committee for Animal Experiments of the Vrije Universiteit Brussel (Lab Accreditation Number: LA1210220).

## Author Contributions

FZ, SS, SM, ND, JV, GR, and YW designed the experiments. FZ and JZha performed the characterization experiments. YW, ZO, JZho, and XW performed crystallography experiments. YW and FZ analyzed data. FZ and YW wrote the manuscript. SS, JV, SM, and GR made manuscript revisions. All authors contributed to the article and approved the submitted version.

## Conflict of Interest

The authors declare that the research was conducted in the absence of any commercial or financial relationships that could be construed as a potential conflict of interest.
