# Supplementary material for: Development and Characterization of Nanobodies Targeting the Kupffer Cell
Source: Front Immunol. 2021 Feb 22;12:641819. doi: 10.3389/fimmu.2021.641819 (PMC7937711; doi:10.3389/fimmu.2021.641819)
Supplement: Supplementary file 1 [file Table_1.DOCX]

**Supplementary data:**

**Supplementary Table1.** X-ray data collection and refinement statistics. Statistics for the highest resolution shell are shown in parentheses.

|  | **Nb1.46** | **Nb2.22** |
| --- | --- | --- |
| **Data collection：** | | |
| Spacegroup | P4_1_2_1_2 | P4_2_ |
| a, b, c (Å) | 73.5, 73.5, 94.8 | 80.5, 80.5, 107.7 |
| α, β, γ (°) | 90, 90, 90 | 90, 90, 90 |
| Resolution (Å) | 45.59-1.98 (2.05-1.98) | 39.12-2.70 (2.80-2.70) |
| *R*merge | 0.077(1.569) | 0.2262 (1.414) |
| *R*meas | 0.079 (1.62) | 0.2584 (1.612) |
| Multiplicity | 24.0 (16.3) | 3.9 (4.1) |
| CC(1/2) | 0.998 (0.669) | 0.981 (0.533) |
| CC* | 1 (0.896) | 0.995 (0.706) |
| I/σ(I) | 23 (1.3) | 5.74 (1.0) |
| Completeness (%) | 99.71 (97.64) | 98.10 (98.25) |
| Wilson B-factor (Å^2^) | 51.56 | 52.67 |
| **Refinement：** | | |
| Total Reflections | 447389 (28937) | 73168 (7662) |
| Unique Reflections | 18661 (1776) | 18555 (1855) |
| *R*_work_/*R*_free_ | 0.1997/0.2165 | 0.2090/0.2528 |
| **Number of atoms:** | | |
| Protein | 973 | 3653 |
| Water | 60 | 67 |
| Average B-factor (Å^2^) | 42.99 | 53.18 |
| Protein ADP (Å^2^) | 42.20 | 53.33 |
| **Ramachandran plot:** | | |
| Favored/Allowed (%) | 98.4/1.2 | 97.1/2.9 |
| **Root-Mean-Square-Deviation:** | | |
| Bond lengths (Å) | 0.010 | 0.005 |
| Bond Angle (°) | 1.41 | 0.96 |
| PDB code | 7DJX | 7DJY |

**Supplementary Table2:** Biochemical properties for 25 evaluated Clec4f nanobodies. The Nbs were grouped according to the protein sequence similarity in the complementarity determining region 3. Twenty-four Nbs were successfully re-cloned in pHEN6c vector. The theoretical pI of the nanobodies were calculated from its amino acid sequence. The Nb yield varied from <1 to 40 mg/ liter culture after IMAC.

| **No** | **Name** | **Round** | **Group** | **theoretical**  **pI** | **MW(Da)** | **Total amount**  **(mg/liter)** |
| --- | --- | --- | --- | --- | --- | --- |
| **1** | Nb1.46 | R2-46 | G1 | 6.29 | 14411 | 8.8 |
| **2** | Nb2.22 | R3-22 | G2 | 9.04 | 13874 | 19 |
| **3** | Nb3.60 | R2-60 | G3 | 9.47 | 13126 | 12.5 |
| **4** | Nb4.39 | R2-39 | G4 | 9.39 | 12467 | <1 |
| **5** | Nb5.10 | R3-10 | G5 | 9.30 | 14121 | 8.5 |
| **6** | Nb6.3 | R3-3 | G6 | 9.30 | 12759 | 10.5 |
| **7** | Nb6.4 | R3-4 | G6 | 8.66 | 12861 | 13 |
| **8** | Nb6.18 | R3-18 | G6 | 8.02 | 12894 | 7.5 |
| **9** | Nb6.19 | R2-19 | G6 | 8.02 | 12914 | 37.7 |
| **10** | Nb6.39 | R3-39 | G6 | 9.39 | 12928 | <1 |
| **11** | Nb7.24 | R2-24 | G7 | 8.38 | 13759 | <1 |
| **12** | Nb8.37 | R3-28 | G8 | 9.01 | 14604 | 4.5 |
| **13** | Nb9.15 | R3-15 | G9 | 8.45 | 14299 | 6.5 |
| **14** | Nb10.16 | R2-16 | G10 | 8.00 | 14307 | 40 |
| **15** | Nb10.17 | R3-17 | G10 | 8.00 | 14242 | 25 |
| **16** | Nb10.21 | R2-21 | G10 | 9.23 | 14308 | 28.25 |
| **17** | Nb10.63 | R2-63 | G10 | 8.98 | 13415 | <1 |
| **18** | Nb10.66 | R2-66 | G10 | 8.98 | 14280 | 37.25 |
| **19** | Nb11.16 | R3-16 | G11 | 9.23 | 14687 | 9.75 |
| **20** | Nb11.20 | R2-20 | G11 | 8.44 | 14759 | 22 |
| **21** | Nb11.28 | R3-28 | G11 | 9.01 | 14604 | 4.5 |
| **22** | Nb11.55 | R2-55 | G11 | 9.23 | 14283 | 25 |
| **23** | Nb12.75 | R2-75 | G12 | 9.27 | 14259 | 23.5 |
| **24** | Nb13.12 | R3-12 | G13 | 7.97 | 14757 | 10 |
| **25** | Nb14.70 | R2-70 | G14 | 8.69 | 14138 | 21.5 |

**Supplementary Table 3:** Summary of increasing RU caused by an additional nanobody (2^nd^ Nb) after saturated binding by 1^st^ Nb on Clec4F. The 1st Nb (on the first column) was allowed to bind to immobilized Clec4F until reaching the epitope saturation. Following the second injection which was a mixture of the 2nd Nb (in the first row) and the 1st Nb. The extra increase in resonance units were obtained by subtracting the RU of the first injection from the RU obtained after the second injection. An increase in RU values of less than caused by binding of the second Nb (10 RU/70 RU = 14%), indicating that these Nbs inhibit each other’s binding and thus seem to share a similar epitope, is indicated in red, whereas RU increase values between 10 and 20 (14% to 28%) are indicated in blue RU increase values between 20 and 40 (28% to 57%) are indicated in yellow. Those values indicated in blue and yellow suggest a partial inhibition between the two Nbs. Finally, RU increase values higher than 40, indicating the absence of inhibition, are shown in black color. An RU value increase of <10 is labeled in red, <20 is labeled in blue, and <30 is labeled in orange.

| **1st\2nd Nb** | **Nb2.22** | **Nb3.60** | **Nb6.19** | **Nb7.27** | **Nb8.37** | **Nb9.15** | **Nb10.16** | **Nb11.16** | **Nb13.12** | **Nb14.70** |
| --- | --- | --- | --- | --- | --- | --- | --- | --- | --- | --- |
| **Nb2.22** | 2.7 | 62.66 | 72.69 | 45.3 | 75.99 | 50.37 | 37.35 | 75.43 | 76.1 | 78.68 |
| **Nb3.60** | 73.44 | 2.79 | 73.2 | 61.86 | 66.55 | 56.46 | 15.97 | 16.78 | 61.65 | 9.98 |
| **Nb6.19** | 75.04 | 65.07 | 2.86 | 57.37 | 72.73 | 91.78 | 75.18 | 60.19 | 72.14 | 40.36 |
| **Nb7.27** | 70.11 | 60.36 | 72.95 | 2.7 | 16.61 | 4.41 | 65.99 | 61.64 | 3.15 | 46.2 |
| **Nb8.37** | 79.85 | 77.22 | 77.42 | 11.86 | 1.46 | 18.76 | 77.16 | 78.13 | 6.26 | 41.86 |
| **Nb9.15** | 64.88 | 71.81 | 56.39 | 6.88 | 2.74 | 2.09 | 67.24 | 67.23 | 14.82 | 28.95 |
| **Nb10.16** | 62.54 | 5.25 | 63.07 | 62.33 | 62.42 | 63.4 | 1.52 | 2.18 | 61.74 | 12.53 |
| **Nb11.16** | 78.09 | 8.94 | 57.82 | 46.31 | 82.55 | 79.63 | 11.98 | 0.09 | 77.92 | 26.65 |
| **Nb13.12** | 73.35 | 75.86 | 75.28 | 13.16 | 4.35 | 5.45 | 74.69 | 72.18 | 4.13 | 73.11 |
| **Nb14.70** | 59.68 | 19.86 | 55 | 46.2 | 14.58 | 58.03 | 33.18 | 3.73 | 57.61 | 2.89 |
